# Risk and protective factors associated with mental health among female military veterans: results from the veterans’ health study

**DOI:** 10.1186/s12905-021-01181-z

**Published:** 2021-02-08

**Authors:** Richard E. Adams, Yirui Hu, Charles R. Figley, Thomas G. Urosevich, Stuart N. Hoffman, H. Lester Kirchner, Ryan J. Dugan, Joseph J. Boscarino, Carrie A. Withey, Joseph A. Boscarino

**Affiliations:** 1 Department of Sociology, Kent State University, 215 Merrill Hall, 700 Hilltop Drive, Kent, OH 44242-0001 USA; 2 Department of Population Health Sciences, Geisinger Clinic, 100 N. Academy Avenue, MC 44-00, Danville, PA 17822 USA; 3 Tulane Traumatology Institute, Tulane University, 127 Elk Place, New Orleans, LA 70112 USA; 4 Ophthalmology Service, Geisinger Clinic, 126 Market Way, Mount Pocono, PA 18344 USA; 5 Department of Sleep Medicine, Geisinger Clinic, 100 N. Academy Ave, Danville, PA 17822 USA; 6 Clinical Psychology Department, William James College, 1 Wells Ave, Newton, MA 02459 USA

**Keywords:** Female veterans, PTSD, Depression, Suicidal ideation, Service use, Post-deployment

## Abstract

**Background:**

This study focuses on factors that may disproportionately affect female veterans’ mental health, compared to men, and is part of a larger study assessing the prevalence of mental health disorders and treatment seeking among formerly deployed US military service members.

**Methods:**

We surveyed a random sample of 1,730 veterans who were patients in a large non-VA hospital system in the US. Based on previous research, women were hypothesized to be at higher risk for psychological problems. We adjusted our results for confounding factors, including history of trauma, childhood abuse, combat exposure, deployments, stressful life events, alcohol misuse, psychological resources, and social support.

**Results:**

Among the veterans studied, 5% were female (n = 85), 96% were White (n = 1,161), 22.9% were Iraq/Afghanistan veterans (n = 398), and the mean age was 59 years old (SD = 12). Compared to males, female veterans were younger, unmarried, college graduates, had less combat exposure, but were more likely to have lifetime PTSD (29% vs. 12%.), depression (46% vs. 21%), suicidal ideation (27% vs. 11%), and lifetime mental health service use (67% vs. 47%). Females were also more likely to have low psychological resilience and to have used psychotropic medications in the past year. Using multivariate logistic regression analyses that controlled for risk and protective factors, female veterans had greater risk for lifetime PTSD, depression, suicidal thoughts, and for lifetime use of psychological services, compared to males. Since 95% of the population in this study were male and these results may have been statistically biased, we reran our analyses using propensity score matching. Results were consistent across these analyses.

**Conclusion:**

Using a sample of post-deployment veterans receiving healthcare services from a large non-VA health system, we find that female veterans are at greater risk for lifetime psychological problems, compared to male veterans. We discuss these findings and their implications for service providers.

## Background

Currently, women are one the fastest growing demographic groups in the military, and the proportion of female military service members and veterans is at its highest level ever in the United States (US) and other industrialized countries [1, 2]. Although women currently comprise only 17% of US active-duty forces, and about 10.5% of current veterans, this percentage is expected to grow. Growth is even greater in the National Guard/Reserve component of the US military. Given current trends, by 2042, women will comprise over 16% of the total US veteran population [1]. These trends are even more pronounced in other advanced industrial countries [3, 4]. Thus, it is critical that we conduct research on factors affecting the well-being of women serving in the armed forces. The goal of this study is to assess both military deployment factors and post-deployment experiences that may contribute to lifetime psychological disorders, especially posttraumatic stress disorder (PTSD) in female veterans, relative to their male counterparts, to optimize future training and treatment planning.

Based on previous research, we hypothesize that female veterans receiving healthcare will have higher rates of PTSD and other mental health problems, compared to male veterans. This hypothesis is tentative, however, as research on sex differences on veterans’ mental health is inconsistent. Some research reports similar combat experiences and stress exposures for men and women among active duty military personnel, as well as commensurate rates of mental health problems [5, 6]. Conversely, other research shows that women’s military experiences and their responses are often different from men’s, placing them at higher risk for psychological problems [3, 7–9]. For example, women experience significantly more sexual harassment and sexual assault prior to and during military service [3, 10]. In addition, some research finds that male veterans are at greater psychological risk for mental health problems [11]. Possible reasons for these sex differences include exposure to different types of trauma, genetics, emotional learning, gender socialization, and memory processing [2]. Our analysis examines if (1) trauma experiences (both military and non-military) and psychological well-being differ between male and female veterans, (2) if we can explain the differences in well-being using multivariate statistical analysis controlling for confounding and other risk factors, and 3) examine sex differences in treatment seeking.

Many of the inconsistent results related to sex differences in US veterans are also seen in studies from other industrialized countries. In their study of Canadian veterans, Brunet et al. report that females were less likely to experience combat related traumas, but more likely to suffer from sexual assaults compared to male Canadian veterans [3]. Like some US studies [7], Canadian female veterans were also more likely to meet criteria for PTSD than males. Woodhead et al., in contrast, report few mental health differences between male and female UK veterans, although these results may be due to the relatively fewer women in the study sample [4].

Much of the previous research on deployment and veteran well-being analyze data from veterans seeking services from the US Department of Veterans Affairs (VA) [11–13], or other government-funded healthcare systems [4]. Our sample, in contrast, comes from a community population of veterans receiving healthcare from a large non-VA system. Many veterans in the US do not use VA healthcare services and recent policy changes will likely increase the number of veterans seeking care from other providers in the future [14–16]. It should be noted, however, that many participants in our study also receive healthcare from the VA. Thus, this study provides insight into a population of veterans that may overlap with VA-based samples but is different from those used in previous studies. Seeking treatment outside of traditional military healthcare systems may also inform policy planning in other countries to the extent that their military institutions are undergoing change and veterans are seeking care in the civilian healthcare system.

Finally, in recent years, the growth of women’s veterans service organizations (VSOs) in the US has been extensive. To date, over 150 active women’s VSOs and related auxiliary groups have been identified (https://womenvetsusa.org/about.php). These VSOs report connecting women with active duty, Reserves, and National Guard military service members and their families, and with caregivers, advocates, and with local, state, and federal resources. In the discussion section below, we briefly discuss the potential impact of these VSOs on mental health outcomes for woman. While we do not directly address the impact of these activities have on women veterans, we would expect that they would likely reduce sex differences in well-being and treatment seeking over time.

## Methods

### Procedure

Data were collected via a telephone survey from a sample of community-based US military veterans recruited to assess the health effects of military service [15]. All participants were outpatients of Geisinger Clinic, the largest multi-hospital system in Central and Northeastern Pennsylvania (see: www.geisinger.org), serving more than 3 million residents. Starting in 2007, Geisinger Clinic collected electronic records on veteran status and patients were asked to complete a military registry form (available upon request from corresponding author [JAB] and attached as a Additional file 1). Using these data, participants were randomly selected for the telephone survey using Geisinger’s electronic health record (EHR) system. We also used screener questions at the beginning of the survey to exclude participants who were institutionalized or incapable of completing a 60-min interview due to physical, language, or cognitive impairment and to confirm veteran status. Screener questions were also used to identify participants who met our inclusion criteria: being able to complete the survey in English, being between 18 and 75 years old, and having at least one warzone deployment. After obtaining informed consent, trained interviewers administered a structured diagnostic interview using the WinCati survey system (Northbrook, Illinois. USA) operating on a local area network (LAN) (see Additional file 1). These interviews took place between February 2016 and February 2017. The final sample size for the survey was 1,730, and the survey cooperation rate was estimated to be approximately 55% [17]. The average time for participants to complete the survey was about 65 min. All participants were offered a $30 incentive for participation. The Institutional Review Boards of Geisinger (IRB #2015-0441) and the Department of Defense (IRB #A-18989) reviewed and approved the study protocols. Since, apart from the demographic, service use, and screener questions used in the study, many of the scales used were proprietary, we detail the instruments used below in the methods section. However, the demographic, service use, and screener questions used in the survey are available as Additional file 2 associated with this article or from the corresponding author (JAB). Noteworthy is that since this was a complex diagnostic interview [16], study surveyors used the “WinCati” system (https://www.sawtooth.com/index.php/software/wincati/), which keeps track of the survey responses and administers the survey electronically, which is a common research practice [17]. Those interested in the veteran-related trauma and social support scales used, should contact the VA to get permission for use and to download these scales (https://www.ptsd.va.gov/professional/assessment/list_measures.asp). Those interested in the depression scale used should go to: https://www.apa.org/depression-guideline/patient-health-questionnaire.pdf to get permission to use and to download this scale from the APA. Those interested in the “Audit-c” scale should go to: https://www.hepatitis.va.gov/alcohol/treatment/audit-c.asp to get permission and to download this scale from the VA.

### Dependent variables

The current study focused on four main outcome variables: PTSD, depression, suicidal ideation, and use of mental health services. To assess lifetime and past year PTSD, we used a diagnostic instrument—the PTSD Checklist based on the Association’s *Diagnostic and Statistical Manual of Mental Disorders*, 5th Edition (*DSM*-5) [18]. To receive a diagnosis of PTSD, veterans had to meet the DSM-5 diagnostic criteria A through G: trauma exposure (criterion A), intrusive symptoms (criterion B), persistent avoidance (criterion C), negative alterations in cognitions/mood (criterion D), increased arousal (criterion E), and reported impairment/distress related to these symptoms (criterion G) [18]. Nearly 80% of the veterans in the current study reported that the most significant lifetime stressor they experienced was warzone or combat exposure [16]. Lifetime and past year depression were measured using a 10-item version of the Structured Clinical Interview for DSM (SCID) Major Depressive Disorder used in previous studies [15, 16]. Consistent with DSM-IV, respondents met criteria for depression (Cronbach’s Alpha = 0.87), if they had 5 or more depression symptoms for at least 2 weeks [19, 20]. We also used a measure of lifetime suicidal ideation to focus more specifically on this health outcome that was used in previous research [14]. Specifically, the item asked if respondents had ever had thoughts for two weeks or longer about how they would be better off dead or “of hurting yourself in some way.” Lastly, the survey inquired about the use of eight different mental health service providers (psychiatrist, general practitioner, psychologist, counselor, spiritual advisor, social worker, or other types of health professional or self-help group) for problems with emotions, nerves, or use of alcohol or drugs. Use of any of these services over the past year or over the lifetime were coded “yes” or “no”, respectively. As with our other measures, these were used in previous studies [14–16] and are available from the corresponding author (JAB) and are attached as an Additional file 2.

### Independent variables

Our survey included demographic, military experiences, recent stressors, social connections, and psychological variables known to affect mental health [14–16]. Demographic variables were sex, age, race, marital status, and education, which were coded such that female, older age (65 +), White race, married (or living as married), and college graduate were coded as the indicator variable. Our military experience variables included deployment era (Iraq/Afghanistan vs. other eras), multiple combat zone tours (coded two or more vs. one), and deployed as National Guard/Reserve or an active-duty service member. We measured combat exposure based on eight items from the Combat Experiences Scale [16, 21, 22]. Several past military health studies used versions of this scale since the Vietnam War period [23]. The 8 items (rated 1 to 4) asked about encountering dead bodies, being wounded by hostile fire, killing enemy combatants, and other combat related events (Cronbach’s alpha = 0.84). We coded the sample into high combat exposure (≥ 75th percentile) versus low exposure. Unit support/morale was based on six items from the Deployment Risk and Resilience Inventory that inquired about a sense of camaraderie in the unit, trust of other unit members, commanding officers being interested in how they felt, feeling like efforts counted in the military, during deployment, etc. (Cronbach’s alpha = 0.78) [24, 25]. We coded respondents into those who reported higher support and unit morale versus those who did not using the scale’s 25th percentile. Lastly, concussion history was assessed based on reported concussions experienced during military service [16]. We note that all these measures are considered important dimensions of military service in combat zones [15, 16] and are available from the VA as noted.

The analysis also included three measures of stress based on previous work [14, 16]. Stressful events in the past year was the sum of 8 experiences (e.g., spouse/mate die, serious injury, problems at work, etc.), which has been used in past research [16]. Lifetime trauma was the sum of 12 experiences (e.g., natural disaster; being attacked with gun, knife, or weapon; being in a situation where they could be seriously injured or suffer physical harm; forced sexual contact, etc.) that could have happened to the respondent in their lifetime [16]. For this trauma assessment, interviewers specifically noted that being “attacked” included not being in combat, since this was assessed separately in the combat exposure scale. Finally, the Adverse Childhood Experiences (ACE) measure was the sum of 12 events (e.g., parent swear at or insult you; parent push, grab, slap, or push you) that could have happened to the participants (never, sometimes, often, very often) before they were 18 [26]. This measure of childhood abuse and neglect has been used in many studies, showing good validity and reliability [26, 27]. For all three of these stress measures, we divided the sample into low versus high exposure [16].

Lastly, we included several psychological, social, and physical health factors that could help explain sex differences in well-being. Psychological resilience was assessed by the 5-item version of the Connor-Davidson Resilience Scale [28], with respondents who fell below the 25th percentile defined as having low resilience [15]. The Cronbach’s alpha for this scale was 0.99. The social support scale (e.g., someone available to help you if you were confined to bed) used in this study was based on four questions (Cronbach's alpha = 0.84) that inquired about emotional, informational, and instrumental support, coded 1–4 (“none of the time” to “all the time”) [29]. This scale has been used in previous trauma studies and is considered a reliable and valid measure of current social support [15, 30–32]. Low social support was defined as cases falling at or below the 25th percentile [30]. Self-rated physical health was assessed using one survey item (fair/poor vs. good to excellent). The survey inquired about past year alcohol misuse, which we operationalized using the three-item Alcohol Use Disorders Identification Test (AUDIT-C), coded positive for misuse for respondents scoring 4 + if male or 3 + if female and self-esteem, using 5-items from Rosenberg’s Self-Esteem scale (Cronbach’s alpha = 0.87), which we divided into low versus high categories, using the 75th percentile as the cut-point. These measures were also used in other studies and show good validity [15, 16].

Finally, we assessed several variables related to health services use. VA service use was assessed using single item questions inquiring about current and lifetime use of VA healthcare service. We also used a single item to ask about current VA disability status (yes vs. no). In addition, the survey asked about the use of psychotropic medications in the past year: anti-depressants, tranquilizers, sleeping pills or other medicines for problems with emotions, nerves, concentrating, sleeping or coping with stress over the past 12 months. Any reported use of these medications was coded yes or no, based on whether the participant used any of these medications in the past year. Thus, this medication measure represented the percent of those that used these drugs in the past year. All these measures were used in previous studies [15, 16] and are available from the corresponding author (JAB), in the attached Additional file 2, from the VA, and from the APA, as noted above.

### Analytic strategy

We present descriptive statistics and bivariate differences between male and female veterans (Table 1). Given the number of females in our sample (n = 85), we conducted preliminary analyses focused on lifetime disorders and retained variables that predicted these outcomes using multivariate logistic regression (Table 2). All variables in the multivariate models had complete data, except for age which had two missing values. We dropped these cases from these analyses. To further examine the relationship between gender and our outcomes, we performed propensity score matching at 1:1, 1:3, and 1:5 ratios of female to male using “nearest neighbors” methods and compared these results from propensity matched cohorts to conventional multivariate logistic regression [33].

**Table 1:** Demographic, deployment, and well-being measures for total sample and by sex (N = 1727–1730)

Study variables	(N)	% Total	Sex			
			%Male	%Female	χ^2^	p-value
Age: 18–64	(751)	43.5	40.8	95.3	97.75	< 0.001
White Race	(1655)	95.7	95.9	90.6	5.56	0.018
Married	(1340)	77.5	78.8	50.6	36.96	< 0.001
College Graduate or Higher	(429)	24.8	23.6	47.1	23.56	< 0.001
Iraq/Afghanistan Veteran	(396)	22.9	21.3	54.1	49.39	< 0.001
Multiple Tours	(686)	39.7	40.3	29.4	3.97	0.046
Deployed as Guard/Reserve	(665)	38.4	37.0	65.9	28.45	< 0.001
High Childhood Abuse/Neglect	(288)	16.6	16.5	20.0	0.72	0.395
High Combat Exposure	(408)	23.6	24.7	2.4	22.36	< 0.001
Low Unit Support	(364)	21.0	20.5	31.8	6.19	0.013
High Stressful Events Past Yr	(375)	21.7	21.3	29.4	3.15	0.080
High Lifetime Trauma	(357)	20.6	20.6	21.2	0.90	0.891
Low Psych Resilience	(439)	25.4	24.3	45.9	19.85	< 0.001
Low Current Social Support	(314)	18.2	17.9	23.5	1.74	0.187
Fair/Poor Current Health	(633)	36.7	37.2	26.2	4.16	0.041
Concussion in Service	(491)	28.4	29.1	14.1	8.95	0.003
Positive Score Audit-C	(109)	6.3	6.4	4.7	0.39	0.535
Low Self-Esteem	(400)	23.1	22.7	30.6	2.80	0.094
PTSD Past Year	(132)	7.6	7.3	14.1	5.34	0.021
PTSD Lifetime	(216)	12.5	11.6	29.4	23.44	< 0.001
Current Depression Disorder	(143)	8.3	7.8	17.6	10.38	0.001
Lifetime Depression Disorder	(381)	22.0	20.8	45.9	29.63	< 0.001
Recent Suicidal Thoughts	(94)	5.2	5.2	9.4	2.75	0.133
Lifetime Suicidal Thoughts	(196)	11.3	10.5	27.1	22.02	< 0.001
Currently Using VA Services	(864)	49.9	50.3	43.5	1.47	0.225
Current VA Disability	(629)	36.4	37.0	24.7	5.25	0.022
Use Psych Services Past Yr	(406)	23.5	22.4	43.5	20.03	< 0.001
Lifetime Use Psych Services	(832)	48.1	47.1	67.1	12.88	< 0.001
Use Psychotropics Past Yr	(384)	22.2	21.4	37.6	12.36	< 0.001
N (%)			1645(95.1)	85(4.9)

**Table 2:** Odds ratios and 95% confidence intervals for mental health outcomes regressed on demographic, deployment, drinking, and psychological resource variables (N = 1728)

Independent variables	Lifetime PTSD OR (95% CI)	Lifetime depression OR (95% CI)	Lifetime suicidal thoughts OR (95% CI)	Lifetime psych services OR (95% CI)
Sex (Female)	5.28 (2.67–10.46)***	3.09 (1.74–5.48)***	2.59 (1.38–4.87)**	1.72 (1.01–2.94)*
Age (65 +)	0.76 (0.51–1.11)	0.51 (0.38–0.68)***	0.73 (0.51–1.04)	0.53 (0.42–0.67)***
College Graduate or Higher	0.84 (0.55–1.29)	0.81 (0.58–1.13)	1.01 (0.68–1.50)	0.95 (0.74–1.22)
Married	1.48 (0.96–2.24)	1.05 (0.75–1.46)	1.22 (0.82–1.82)	0.66 (0.51–0.87)**
High Child, Abuse/Neglect	1.67 (1.12–2.48)*	2.13 (1.54–2.95)***	2.25 (1.57–3.24)***	1.87 (1.38–2.55)***
High Stress past Yr	3.30 (2.28–4.78)***	2.27 (1.67–3.08)***	1.22 (0.84–1.79)	2.06 (1.55–2.75)***
High Lifetime Trauma	2.36 (1.63–3.41)***	1.64 (1.20–2.24)**	1.20 (0.82–1.76)	1.54 (1.16–2.05)**
High Combat Exposure	3.07 (2.07–4.54)***	1.86 (1.35–2.58)***	1.21 (0.82–1.80)	1.69 (1.29–2.22)***
Concussion in Service	2.31 (1.59–3.36)***	1.50 (1.10–2.05)**	1.13 (0.77–1.64)	2.05 (1.59–2.66)***
Positive AUDIT-C	1.59 (1.09–2.34)*	1.07 (0.78–1.46)	1.05 (0.72–1.52)	1.08 (0.83–1.39)
Low Self-Esteem	3.03 (2.07–4.44)***	3.40 (2.51–4.62)***	4.25 (2.94–6.15)***	2.37 (1.76–3.17)***
Low Resilience	2.24 (1.53–3.28)***	2.32 (1.72–3.15)***	1.95 (1.35–2.81)***	2.02 (1.53–2.67)***
Low Current Social Support	1.69 (1.12–2.55)*	1.62 (1.16–2.27)**	1.34 (0.91–1.98)	0.94 (0.70–1.27)
Constant	0.009***	0.075***	0.034***	0.655*

Logistic Regression:OR-Odds Ratio CI-Confidence Interval

Significance levels:* p < .05 * p < .01*** p < .001

### Propensity score matching

For propensity score matching we first included the confounding covariates listed in Table 2, including age, college graduate, married status, high combat exposure, serving on multiple tours, low psychological resilience, high neglect/abuse history, high current life stress, high lifetime trauma, history of concussions, low self-esteem, and low social support. In addition to these variables, we also added reported history of ADHD (a doctor told respondent he/she had this disorder), Iraq/Afghanistan military service, low unit support during deployment, based on the DRRI scale [24, 25], military rank (officer vs enlisted), White race, assessment of “stable emotions,” based on the 5-factor personality scale [34], and current reported VA service use to estimate the propensity score for female sex. Then, our matching procedure was executed using 1:1 nearest neighbor matching without replacement where a single female participant was matched to a single male participant who had the most similar estimated propensity score with a caliper of 0.2. In addition, as the sample sizes of the female and male participants varied greatly, we performed the one-to-many matchings where a single female participant was matched to more than one male participant (e.g., 1:3 and 1:5 matching) using nearest neighbor method based on propensity scores [33]. There were 85 females and 1644 males in original dataset. After propensity score matching, there were 85 females and 85 males selected for the 1:1 matching; 85 females and 255 males selected for the 1:3 matching; and 85 females and 425 males selected for the 1:5 matching. Multivariate logistic regression models were then conducted for the 1:1, 1:3 and 1:5 nearest neighbor matching to evaluate the sex differences in predicting lifetime PTSD, depression, suicidal thoughts, and lifetime use of psychological services (Table 3). The propensity scores matching procedures were conducted in RStudio Version 1.2.1335, the “MatchIt” package [35, 36]. As a further test of model adequacy, we performed a sensitivity analysis using the Wilcoxon Signed Rank Test, where the value of Gamma can be interpreted as the odds of the model suffering from hidden bias due to omitted variables [37].

**Table 3:** Multivariate logistic regression results using propensity score matching for lifetime PTSD, depression, suicidal Ideation, and lifetime psychological services

Dependent variable	OR (95% CI)	Pr( >|z|)
*Matching: PTSD*		
1 to 5 female versus male	5.19 (2.43 11.09)	0.00002***
1 to 3 female versus male	5.13 (2.32 11.34)	0.000523***
1 to 1 female versus male	11.55 (3.06 43.63)	0.000309***
*Matching: Major Depression*		
1 to 5 female versus male	3.27 (1.77 6.06)	0.000162***
1 to 3 female versus male	3.04 (1.61 5.74)	0.000635***
1 to 1 female versus male	2.64 (1.19 5.84)	0.0171*
*Matching: Suicidal Ideation*		
1 to 5 female versus male	2.48 (1.30 4.75)	0.006106**
1 to 3 female versus male	2.82 (1.41 5.63)	0.003367**
1 to 1 female versus male	3.99 (1.53 10.37)	0.004533**
*Matching: Psych Services*		
1 to 5 female versus male	1.91 (1.09 3.38)	0.024818*
1 to 3 female versus male	1.96 (1.08 3.57)	0.027033**
1 to 1 female versus male	1.46 (0.71 2.99)	0.299672

Significance levels: *** < 0.001 ** < 0.01 * < 0.05

## Results

The basic characteristics of the sample (Table 1) show that 5% (n = 85) were female. Other features of the sample show numerous large differences between male and female veterans. For example, about 95% of the females were less than 65 years old, while only about 40% of the male veterans were in that age range. About half the women were married, but almost 80% of the men were, and female veterans were also more likely to be college graduates, compared to their male counterparts (47% vs. 24%). Female veterans were more likely to see service during the Afghanistan/Iraq War (54% vs. 21%), be deployed in National Guard/Reserve units (66% vs. 37%) and were much less likely to report high combat exposure (2% vs. 25%), compared to men. The sample shows smaller, but statistically significant differences for race, multiple tours, and unit support, with women being more racially diverse, fewer reporting multiple tours, and higher percentages scoring low on unit support/morale. Data also revealed psychological and health differences between female and male veterans (Table 1). Women were more likely to score low on psychological resilience, less likely to report service-related concussions, and more likely to report poor health. Interestingly, there were no sex differences for childhood abuse/neglect, reported stressful events in the past year or lifetime traumas, social support, alcohol misuse, or self-esteem.

Female veterans in our study were more likely to meet criteria for lifetime and past year PTSD, meet criteria for lifetime and past year depression, and lifetime suicidal thoughts, but not recent ones. Health service results show no sex differences in the current use of VA services, but women were less likely to report a current VA disability, and more likely to have used any (VA or non-VA) psychological services and psychotropic medications in the past year.

Multivariate logistic regression results (Table 2) revealed that women were more likely to meet study criteria for lifetime PTSD (OR = 5.28), depression (OR = 3.09), suicidal thoughts (OR = 2.59), and more likely to report lifetime use of psychological services (OR = 1.72), after adjusting for other demographic factors, stressful events, alcohol misuse, psychological resources, and social support. Other statistically significant factors predictive of lifetime PTSD were childhood adversities (OR = 1.67), past year stressors (OR = 3.30), lifetime trauma (OR = 2.36), combat exposure (OR = 3.07), concussion history (OR = 2.31), positive AUDIT-C results (OR = 1.59, low self-esteem (OR = 3.03), low psychological resilience (OR = 2.24), and low social support (OR = 1.69).

In addition to sex, age was statistically related to lifetime depression (OR = 0.51), as were childhood adversities (OR = 2.13), stressful events (OR = 2.27), lifetime trauma (OR = 1.64), combat exposure (OR = 1.86), concussion history (OR = 1.50), low self-esteem (OR = 3.40), low resilience (OR = 2.32), and low social support (OR = 1.62). The model for lifetime suicidal thoughts showed that childhood adversities (OR = 2.25), low self-esteem (OR = 4.25), and low resilience (OR = 1.95), along with sex, were statistically related to this psychological problem. Finally, besides statistically significant differences by sex, the model for lifetime psychological service use showed that child abuse (OR = 1.87), high stress in the past year (OR = 2.06), high lifetime trauma (OR = 1.54), high combat exposure (OR = 1.69), service related concussion (OR = 2.05), low self-esteem (OR = 2.37), and low resilience (OR = 2.02) were associated with a higher likelihood of service use, while being older than 65 (OR = 0.53) or married (OR = 0.66) was related to a lower likelihood.

As an additional check on sex differences in well-being among our sample of veterans, we replaced lifetime outcomes with current measures of each variable. That is, we replaced lifetime PTSD, depression, suicidal thoughts, and use of psychological services, with PTSD past year, current depression, recent suicidal thoughts, and use of services in the past year. As shown in Table 1, statistically significant bivariate sex differences were found for PTSD past year, current depression, and psychological service use in the past year, with female veterans more likely to have these mental health problems and to report the use of psychological services. When we controlled for the same factors in multivariate models shown in Table 2, sex differences remained for PTSD (OR = 2.53, p = 0.036) and for use of psychological services (OR = 2.43, p = 002), but not for current depression (OR = 1.65, p = 0.202) or suicidal ideation (OR = 1.09, p = 0.856). We also estimated multivariate models with interaction terms for gender and the stress variables (i.e., child abuse, stress past year, lifetime trauma, and combat exposure) to see if women responded differently to these events relative to men, as some have suggested [2]. None of these interaction terms were statistically significant. (Results are available from the corresponding author.)

In Table 3, multivariate logistic regression results were presented for the 1:1 matching, 1:3 and 1:5 propensity score matching using “nearest neighbor” statistical methods, as discussed above, to evaluate the odds ratio of sex differences in predicting lifetime PTSD, lifetime depression, and lifetime suicidal thoughts. As can be seen, these are all statistically significant, except for 1:1 matching for lifetime use of psychological services. The differences in the number of cases used in the matching are likely a reason for the divergent statistical findings reported in the table. Nevertheless, these results add strength to our conclusions about the differences between male and female veterans for the study outcomes. That is, matching male and female veterans using propensity scores showed that female veterans were five times more likely to meet criteria for PTSD, and two and a half times at greater risk for major depression and suicidal ideation, and more likely users of psychological services compared to male veterans. As a further test of our model, we conducted a sensitivity analysis which showed that the values of Gamma were close to 1 for lifetime PTSD, depression, and suicidal ideation, which suggests that these models may have unmeasured confounders [37]. (Results available from the corresponding author [JAB].) We discuss these limitations in the study conclusion.

## Discussion

Using data collected from a sample of post-deployment veterans receiving their care from a non-VA healthcare system, we assess sex differences in health outcomes. Regarding our three research questions, we find significant sex differences in military and non-military experiences, with female veterans less likely to experience high combat and in-service concussion, but more likely to perceive low unit support. Women veterans were also more likely to meet criteria for lifetime and past year PTSD, lifetime depression, and lifetime suicide ideation. They are more likely to report using mental health services in their lifetime and in the past year. These sex differences in health outcomes persisted even after controlling for other variables. Our study is consistent with earlier research which finds female veterans at greater risk for lifetime PTSD and other mental health problems, and higher users of psychological services [2, 5, 6, 9, 11, 18]. We further checked our findings by presenting propensity score and sensitivity analyses. All are consistent in supporting our conclusions. Our findings are especially important in that women are at greater risk for lifetime suicidal ideation and for the use of psychological services, both lifetime and in the past year. Finally, in line with other research, women in our study were different from male veterans in that they were younger, more educated, less likely to be married, and more likely to be deployed as part of the National Guard/Reserve, consistent with other studies of US veterans [6, 7, 11], as well as studies of veterans who served in other countries [4].

Past research by Lehavot et al. analyzed data from the 2012–2013 National Epidemiologic Survey on Alcohol and Related Conditions-III and, like our findings, report that women veterans have the highest rates of lifetime and past year PTSD and treatment utilization, compared to male veterans [2], and that controlling for different types of trauma (e.g., early childhood abuse, interpersonal violence, and recent stressful events) reduced sex differences, but did not eliminate them [39]. Using VA administrative data, Haskell and her colleagues find that female veterans have greater mental health problems, such as depression and adjustment disorder, and service use, compared to men, but, in contrast to our study, male veterans had higher rates of PTSD, after adjusting for demographic differences [11]. To our knowledge, though, ours is one of the first studies to report findings for female veterans seen in non-VA hospitals [14]. Clinicians in both VA and non-VA facilities need to be aware of demographic, pre- and post-deployment experiences, and the needs of female veterans. Moreover, in their study of women who use the VA for mental health care, Kimerling et al. report that only half of the female veterans found that the VA met their needs [40]. Given the increasing concern among the Department of Veterans Affairs and health policy planners about the rise in suicide rates among veterans [7], our findings strongly argue for more research on unmet needs among female veterans using non-VA facility data to ensure that this population does not suffer from the lack of appropriate care.

There are two points about our results that should be noted, one related to the propensity score and sensitivity analyses and one on the sex differences observed in the lifetime and past year psychological service use. The logistic regression models for our four outcomes included demographic, military experiences, and non-military experiences variables. Nevertheless, all four of the models continued to show sex differences. The propensity score analysis confirmed these results, but the sensitivity analysis indicated the possibility of omitted factors in the models for PTSD, depression, suicidal thoughts, and use of psychological services. Research shows that female veterans not only have different backgrounds (e.g., are more educated and less likely to be married), but also have different life experiences (e.g., more likely to be sexually harassed) than male veterans [2, 5, 6, 9, 41]. The result of failing to capture these differences in our models is that we continue to see statistically significant differences for sex. A model that adequately assess these experiences should show no or few statistically significant differences for sex. Future research should carefully consider how pre-military experiences, self-perceptions, and interactions with others affects men and women differently, especially within a military context.

The statistical significance for sex in the models for lifetime and past year use of psychological services also suggests the need for an examination of the differences in male and female veteran experiences. It is noted that in the lifetime model, the odds ratio for the logistic regression reached statistical significance, and the propensity score results showed statistically significant sex differences for the 1:3 and 1:5 models. Studies suggest sex differences can influence both the experience of stressful events and how persons respond to such events, including treatment seeking [40]. Further, more attention should be paid to the different military experiences of men and women veterans as they relate to these factors. In our study, for example, men were more likely to be deployed to Vietnam, while women were more likely deployed to Iraq/Afghanistan. Women were much more likely to be deployed as National Guard/Reserve than men, and more likely to have had low psychological resilience. Women were also significantly younger in our study. The fact that sex differences persist even after including many controls suggest that sex differences play a key role in mental health outcomes and use of psychological services. Greater attention to these social and psychological sex differences, along with other factors, may provide greater insight onto the psychological consequence of military deployments and may help identify gender gaps in services.

In terms of study limitations, our data were cross-sectional, which precludes assessment of causality. Second, we only were able to successfully recruit 85 female veterans. As noted, this may have biased our results and may not represent the larger population of female veterans receiving care. Although our propensity score analysis confirmed the multivariate analyses, it is possible that unmeasured variables, such as a more detailed history of sexual assault, might change our results. It is also possible that only certain types of female veterans seek healthcare in non-VA facilities, and we have not included this confounding factor in our models. Future research should sample veterans receiving care from both VA and non-VA facilities to broaden generalizations for this population. Third, the current study only included previously deployed U.S. veterans seen at a large non-VA multihospital system in Pennsylvania and our results may not generalize to all veterans. Research which includes veterans from both VA and non-VA systems may better clarify gaps in care. Fourth, the findings may not generalize to non-White US veterans because over 90% of our sample was White. Finally, we did not ask about gender identity, sexual assault/harassment in the military, or sexual orientation. Future studies should explore these issues in more detail, as these have been related to poor mental and physical outcomes among veterans [41]. Many of these limitations are also found in other studies, especially for those of veterans who served in industrialized countries other than the US [4–7, 38–40].

In recent years, the growth of Veteran Service Organizations (VSOs) for women has been extensive. While outcome data related to VSOs are limited, preliminary studies are encouraging [42–44]. For example, while these VSOs appear to be well received, several studies suggest that the more the veteran’s VSO engagement, the larger the meetings, the more involvement in activities, the better are the outcomes for women veterans [42–44]. These VSO findings are promising, although further research is required to better assess the VSO’s impact on women’s mental health.

## Conclusions

Despite these limitations, our findings support the case for more gender-informed planning, given the projected increase in female veterans in both the US and other industrial countries. These changes might include hiring more female healthcare providers, more explicit training in the health needs of female veterans, and training on greater sensitivity to a female’s unique life experiences, such as sexual harassment [1, 40]. Further, and consistent with other researchers [1, 39, 40], we find female veterans have higher use of mental health care services, relative to male veterans, and have found that a higher percent of them accessed psychiatric services in the past year. In the Kimerling et al. study [40], results suggest that female veterans reported lower use of VA healthcare because there were fewer female doctors and women-only healthcare settings. Our findings, along with other studies on female veterans, need to be used to inform changes in the provision of healthcare services to US veterans at both VA and non-VA facilities [15]. Studies of non-VA healthcare service delivery to veterans will be important to develop future public–private partnerships, which will be key in addressing gender differences in the healthcare needs of male and female veterans [40].

## Supplementary information

**Additional file 1.** Veterans registry forms.

**Additional file 2.** Survey script.

## Data Availability

The data used in the current study are not available, since study subjects were patients in a regional healthcare system, and we did not have permission to release these data at this time. The surveys used in the study are posted as supplemental to this article. The scales used in this study are described in the methods section are available from the VA and the APA, as noted in the methods section.

## Abbreviations

PTSD: Posttraumatic Stress Disorder
VA: Veterans Administration
DSM-5: Diagnostic and Statistical Manual, Version 5
SCID: Structured Clinical Interview for DSM
DSM-IV: Diagnostic and Statistical Manual, Version IV
ACE: Adverse Childhood Experiences
VSO: Veterans Service Organization
EHR: Electronic Health Record
