# Supplementary material for: Risk and protective factors associated with mental health among female military veterans: results from the veterans’ health study
Source: BMC Womens Health. 2021 Feb 8;21:55. doi: 10.1186/s12905-021-01181-z (PMC7869200; doi:10.1186/s12905-021-01181-z)
Supplement: Supplementary file 1 — Additional file 1. Veterans registry forms. [file 12905_2021_1181_MOESM1_ESM.pdf]

INTRODUCTION AND CONSENT

Hello, my name is______and I am calling from the Geisinger Center for Health Research in Danville, PA. May I please speak to____?

(IF PATIENT BEING CALLED DOES NOT ANSWER PHONE, STATE FOLLOWING :) I am sorry but I need to speak to Mr./Ms. directly.

(IF PATIENT NOT AVAILABE, STATE:) Is there a better time to call?

(IF YES, WHEN WOULD BE A GOOD TIME TO CALL YOU BACK? SET CALLBACK TIME IF RESPONDENT/PATIENT GIVES YOU A BETTER TIME TO CALL) Date: ____MO_____DAY Time:____AM ____PM

**OK…Thank you for your time today and we will call you back soon.
Have a good day**

(IF RESPONDENT/PATIENT DOESN'T OFFER A CALLBACK TIME, USE DISPOSITION "9" PATIENT NOT AVAILABLE)

(WHEN REACH PATIENT, STATE FOLLOWING)

Hello Mr/Ms_______________, the Center for Health Research is a part of the Geisinger Health System. We are conducting a survey of Geisinger patients who served in the US military to help better understand their healthcare needs. This study is funded by the US Department of Defense (DoD). This Geisinger study is not affiliated with the VA in any way. We mailed out a letter about the study and this phone call. Did you receive the letter?

(IF DID NOT RECALL RECEIVING LETTER, STATE FOLLOWING:) Since you did not receive the letter, may I tell you about the study before you decide to participate in the interview?

(IF YES, CONTINUE BELOW; IF "NOT INTERESTED" IN STUDY, *YOU MAY USE PROTOCOL BELOW WITH RESPONDENT*)

Let me review some of the study details with you. This study is being conducted to understand health issues that may be affecting US Veterans. We are inviting patients 18 years or older, who have served in the US military to take part in this study. Our records indicate you are a US military veteran. Is this correct? (IF THE PATIENT SAYS HE/SHE IS NOT A US VETERAN, THANK THE RESPONDENT AND TERMINATE THE INTERVIEW) I'm sorry. We are interviewing people who are veterans of the US military and have served in a war zone, have seen combat, served in a peace-keeping mission, or served overseas in the Global War on Terror.

Have a nice day.

The study involves a telephone interview, which may take about 50 minutes to complete. We also plan to include data from your Geisinger medical record. The survey will ask about your military experiences and about how you are doing now, your mood, and questions about your mental and physical health status. The information you provide will help us develop better ways to provide care for US veterans. You may decline to answer any questions or end the interview at any time. If you qualify for the study and complete the survey, we can offer you a $20 gift card for you time and effort.

The information you give will be kept confidential, as required under local, state and federal laws. Your individual responses will not be identified, but used together with other patients to provide statistical information for our study.

IF NO, would you like some time to think about it and be called back at a later date? IF YES, arrange call back

IF YES, during your US military service did you ever serve in a war zone, see combat, serve in a peace-keeping mission, or serve overseas in the Global War on Terror?

IF NO, I am sorry but you do not qualify. Thank you for your time and have a nice day.

IF YES, it appears that you do qualify for our interview. In the telephone interview I will ask a few questions that may be sensitive in nature. If you do not feel comfortable answering any of these questions, you do not have to.
Would you like to continue with the interview and participate in the study?

(IF STATES NO/NOT NOW, STATE:) Would you like some time to think about it and be called back at a later date?

Yes (schedule call back below) No
(thank and terminate below)

(IF YES, SET CALLBACK TIME IF RESPONDENT/PATIENT GIVES YOU A BETTER

TIME TO CALL) Date:_____MO_____DAY Time:____AM ____PM

(IF NO, USE THE FOLLOWING:)
Q: RA start:
I am sorry to hear that you are not interested in participating in our survey.
Before you go, I'd like to understand the reason why you are not interested.

1. **Too busy**
2. **Just not interested**
3. **Bad experience w/ Geisinger**
4. **Does not want to give out information over the phone**
5. **Has government, VA, or DoD concerns**
6. **Other: ____________________**

(IF AGREES TO DO INTERVIEW, CONTINUE BELOW:)

Now I will continue with our telephone interview, which will ask a few sensitive questions. If you do not feel comfortable answering any of these questions, you do not have to.

I.1. Would you like to continue with the interview?

Yes (go to Q # I.5) No
(go to Q # I.3)

I.3. Would you like some time to think about it and be called back at a later date? Yes
(schedule call back)
No (go to Decline and terminate)

(IF YES, SET CALLBACK TIME IF PATIENT GIVES YOU A BETTER TIME TO CALL)

Date:__MO____DAY Time:_AM ___PM

DECLINE: I am sorry to hear that you are not interested in participating in our survey. Thank you for your time and have a nice day.

MILITARY HISTORY

1.5 Thank you for agreeing to do this interview. Let's get started

M1. (M.1.1) During your US military service, what war zones did you serve in (READ LIST, RECORD ALL THAT APPLY; IF NO WAR ZONE SERVICE; THANK & TERMINATE INTERVIEW)

In Korea during Korean War, between 1950-1954?..…………………...…..1
In Vietnam during Vietnam War, between 1960-1975? 2
In the Persian Gulf during the Persian Gulf War, between 1990-1992?......3
In Afghanistan or Iraq during Afghanistan/Iraq War, between 2001-2016..4
In other war zones or conflicts, such as Panama invasion, Haiti, Bosnia-Herzegovina, Kosovo, The Sinai,..5
Global War on Terror (2001-2016) 6
Other warzone (RECORD OTHER:________________________.........…7

(NOTE IF ONLY SERVED IN KOREAN WAR, THANK & TERMINATE, NOT QUAIFIED FOR STUDY)

Draft (M1.1.) (IF SERVED IN VIETNAM, PERSIAN GULF, OR OTHER WAR ZONES, ASK:)
Were you ever drafted?

Yes 1
No 2
DK/NA 9

M1a. (IF WAS IN AFGHANISTAN/IRAQ DURING AFGHANISTAN/IRAQ WAR, OR DEPLOYED IN GLOBAL WAR ON TERROR BETWEEN 2001-2016; ASK:)

You mention that you served in Afghanistan or Iraq during Afghanistan/Iraq War or served overseas on the Global War on Terror between 2001-2016 during your US military service. During this service, were you ever in the National Guard or in the Reserves?

National Guard. 1
Reserves.. 2
Both Guard/Reserves... 3
Neither Guard or Reserves. 4
Uncertain. 5

M.2. During your US military service, what service branch did you serve in (REORD ALL THAT APPLY) **Yes** **No**

Air Force
Army Navy
Marine Corps
Coast Guard
Other (SPECIFY:) _______________

M.3. Did you ever serve in the National Guard or the Active Reserves during your US military service **anytime before 2001**?

Yes ……………………………………1
No……………………………………..2

M.4a. (IF YES IN M.4, ASK:) During this time in the National Guard or Reserves, were you ever deployed to a warzone or on a peace-keeping mission?

Yes ……………………………………1
No……………………………………..2

M.4. How many overseas deployments have you served in for 30 days or more that involved service in a warzone?

One
2
3
4
More than 5
DK

M5a. What was your highest military rank?

E1-E3?... 1
E4?.. 2
E5-E7?... 3
E8-E9?... 4
O1-O2?... 5
O3-O4?... 6
O5-O6?... 7
O7-O10?... 8
W1-W2? 9
W3-W5? 10

M.5. Now questions about your health. How would you currently rate your overall physical health -- excellent, very good, good, fair, or poor?

Excellent……………………….……….1
Very Good……………………………….2
Good……………………..……………….3
Fair……………………….……………...4
Poor ……….………….…..…………….…...5
Don‟t Know…………………….……………. 8
Refused……………………….………………9

(Combat Exposure)

M.6. Now some questions about your combat experiences. During your US military service in a war zone or your peace-keeping experiences...

	Never	Rarely	Sometimes	Often
a. Did you encounter dead bodies or see people killed wounded?..	1	2	3	4
b. Did you engage in direct combat where you discharged a weapon?				
c. Did you ever feel that you were in great danger of being killed?				
d. Were you ever wounded by hostile fire?				
e . Were you directly exposed to landmines, mortar, artillery, or rocket fire?				
f. Were you ever stationed at a forward operating combat base?				
g. Did you serve in a combat unit, such in an infantry, armor, cavalry, or an artillery unit?				
h. Were you directly or indirectly responsible for killing enemy combatants?				

(Concussion History)

M.9. During your warzone deployments, do you recall and accident/injury where you experienced…

	YES	NO	DK	REF
a. Being dazed, confused or "seeing stars"?				
b. Not remembering the accident/injury?				
a. Loosing consciousness (knocked out) for a minute or less?				
b. Loosing consciousness for 2-5 minutes?				
c. Loosing consciousness for more than 5-10 minutes?				
d. Loosing consciousness for more than 10 minutes?				

M.9a. Are you currently experiencing any of the following problems [RECORD ALL THAT APPLY]:

	YES	NO	DK	REF
a. Headaches?				
b. Dizziness?				
c. Memory problems?				
d. Balance problems?				
e. Ringing in the ears?				
f. Sleep problems?				

M.10. Throughout our lives, most of us have had pain from time to time, such as minor headaches, sprains, and toothaches). Have you had pain other than these everyday kinds of pain during the past 7 days? YES ....……………. 1

NO ....……………... 2 **[SKIP TO M15]**

DON"T KNOW ....……………..... 8 **[SKIP TO M15]**

REFUSED ....……………. 9 **[SKIP TO M15]**

M.11. Please rate your pain by indicating a number from 0 to 10 that best describes your pain at its worst in the last 7 days, where 0 means no pain and 10 means pain as bad as you can imagine.

_____NUMBER [0-10]

DON‟T KNOW...............................998
REFUSED ....… 999

M.13. Please rate your pain by indicating a number from 0 to 10 that best describes your pain on the average in the last 7 days, where 0 means no pain and 10 means pain as bad as you can imagine.

_____NUMBER [0-10]

DON‟T KNOW...............................998
REFUSED ....… 999

M.14. Now rate your pain by indicating a number from 0 to 10 that tells how much pain you have right now, where 0 means no pain and 10 means pain as bad as you can imagine.

_____NUMBER [0-10]

DON‟T KNOW...............................998
REFUSED ....… 999

M.15. In the past 12 months have you taken prescription painkillers such as codeine, morphine, percodan, viocodin, percocet, hydrocodone, oxycontin, or drugs like these?

YES ....……………. ....1 **(GO TO M15a)**
NO ....……………... 2 **(GO TO M16)**
DON‟T KNOW ....……………....8 **(GO TO M16)**
REFUSED ....……………. 9 **(GO TO M16)**

M15a. About how long **were you** on these medications?

1 WEEK OR LESS
2-3 WEEKS
1-2 MONTHS
3-5 MONTHS
6-9 MONTHS
10-12 MONTHS
1 YEAR OR MORE
DON‟T KNOW
REFUSED

M.16. Next, I am going to read a list of common medical conditions. Please tell me if you have ever been told by a doctor that you had any of these conditions? [READ LIST – MULTIPLE RECORD FOR EACH YES]

Cancer? Stroke?
High blood pressure? Angina?
Heart attack?
Congestive heart failure?
Rheumatoid arthritis? Thyroid disease?
Depression?
Attention deficit disorder or ADHD?
Problems with alcohol or drugs?
Chronic lung disease?
Diabetes?
Asthma?
Ulcers?
Irritable bowel syndrome?
Kidney disease?
Liver disease?
Another mental disorder or mental problem?
[VOL] None of these
[VOL] Refused/Don't know

M.17. During the past 4 weeks, how much did pain interfere with your normal work, including both work outside the home and housework?
Not at all A
Little Bit
Moderately
Quite a bit
Extremely
[VOL] Don‟t know
[VOL] Refused

(SOCIAL SUPPORT)

SS.0. I am now going to ask you some questions about help you have available if you need it. In the past 12 months, how often was each of the following kinds of support available to you if you needed it? In the past 12 months, how often was [ITEM]? Would you say you: [READ CHOICES]?

**SHUFFLE ITEMS**	None of the time	Some of the time	Most of the time	All of the time	DK [VOL]	REF [VOL]
a. Someone available to help you if you were confined to bed?	1	2	3	4	8	9
b. Someone available to give you good advice about a crisis?	1	2	3	4	8	9
c. Someone available to get together with for relaxation?	1	2	3	4	8	9
d. Someone available to confide in or talk about your problems?	1	2	3	4	8	9

SS.2. Do you have a regular doctor or a usual source of health care that you can go to for your routine medical care?

Yes ………………….1
No ………………….2
Don't know VOL]..….8
Refused VOL]………..…9

SS.3. Do you currently have regular health care coverage, including health insurance, prepaid plans such as HMOs, or government plans such as Medicare or Medicaid?

Yes ……………………..1 No
No>……………………..2
Don’t know [VOL]……....8
Refused VOL]..……..…9

SS.3b. Do you currently have any health care coverage or receive health care services through the VA?

Yes ………………………..1 (GO TO SS.3d)
No ………………………..2
Don't know [VOL]……..…8
Refused [VOL]………....…9

SS.3c. Have you ever had any health care coverage or receive health care services through the VA in the past?

Yes.......…………………..1
No ………………………..2
Don't know [VOL].……….8
Refused [VOL]………..…9

SS.3d. Have you ever applied for disability benefits through the VA?

Yes.......…………………...1
No ………………………...2
Don't know [VOL].……….8
Refused [VOL]………..….9

SS.3dd. (IF YES TO SS3.d, ASK:) Do you currently receive disability benefits through the VA?

Yes.......………………..…..1
No ……………………..…..2
Don't know [VOL].……….8
Refused [VOL]………..…9

(Stressful Events)

SE.1. In the past 12 months did any of the following occur… [ITEM]? [DO NOT ROTATE]

-

	Yes	No	Don't know	Refuse
a. Did your spouse or mate die?..........	1	2	3	4

b. Did you get divorced or separated from your mate?……………………	1	2	3	4
c. Did a close family member other than your spouse die?.......................	1	2	3	4
d. Were you seriously injured or seriously ill?.....................................	1	2	3	4
e. Did you get married?.....................	1	2	3	4
f. Did you have family problems with your spouse or children?...................	1	2	3	4
g. Did you have problems at work?..	1	2	3	4
h. Did you have serious financial problems?………………………….	1	2	3	4

(TREATMENTS)

Next, I have a few questions about professional services…

TS.1. Did you ever in your lifetime go to see any of the following professionals or self-help groups for problems with your emotions or nerves or for problems with your use of alcohol or drugs?

	YES	NO	DK	REF
a. A psychiatrist?………………...………………….	1	2	8	9
b. A general practitioner or other medical doctor?…..	1	2	8	9
c. A psychologist?…………………………...……	1	2	8	9
d. A counselor?………………………………………	1	2	8	9
e. A minister, priest, rabbi or other spiritual advisor?.	1	2	8	9
f. A social worker?…………………………………...	1	2	8	9
g. Another type of health professional (SPECIFY:_)?………………………..	1	2	8	9
h. A self-help group, such as Alcoholics Anonymous (AA) or Narcotics Anonymous (NA)?…………….	1	2	8	9

(IF NO TO ALL ITEMS IN TS.1 ABOVE, SKIP TO: TS.4)

TS.2. Over the past 12 months, did you go to see any of the following professionals or self-help groups for problems with your emotions or nerves or for problems with your use of alcohol or drugs?

	YES	NO	DK	REF
a. A psychiatrist?………………...………………….	1	2	8	9
b. A general practitioner or other medical doctor?…..	1	2	8	9
c. A psychologist?…………………………...……	1	2	8	9
d. A counselor?………………………………………	1	2	8	9
e. A minister, priest, rabbi or other spiritual advisor?.	1	2	8	9
f. A social worker?…………………………………...	1	2	8	9
g. Another type of health professional (SPECIFY:_)?………………………..	1	2	8	9
h. A self-help group, such as Alcoholics Anonymous (AA) or Narcotics Anonymous (NA)?…………….	1	2	8	9

TS.3. Over the past 12 months, have you taken medications prescribed by a doctor, such as anti-depressants, tranquilizers, sleeping pills or other medicines for problems with your emotions or

nerves, problems with concentrating, to help you sleep or cope with stress?

Yes……………………………………..……..1
No………………………………………2
Don't know…………………………….…..8
Refuse…………………………………..9

(IF YES TO TS.3, ASK:) TS.3a. Over the past 12 months, on how many days have you taken medications prescribed by a doctor, such as anti-depressants, tranquilizers, sleeping pills or other medicines for problems with your emotions or nerves, problems with concentrating, to help you sleep or cope with stress? (IF SAYS DON'T KNOW, ASKED FOR BEST ESTIMATE)

____ Days [0-365]
DK/Ref

TS.5. Following your war zone **or peace-keeping** service experiences, did you seek help from any of the following to help you with your emotions or nerves, mental health or for problems with alcohol or drugs …

	YES	NO	DK	REF
a. Family and close friends?				
b. Your local neighborhood or community?				
c. Your church or spiritual community?				
d. A clergymen or spiritual advisor?				
e. A chiropractor or acupuncturist?				
f. A herbalist, naturopath or homeopath?				
g. Another nontraditional healer (SPECIFY:_________________________)				

(SUBSTANCE USE)

SU.1. Have you smoked at least 100 cigarettes in your entire life?

1 Yes
2 No [**GO TO SU.6**]
3 [VOL] Don't know [**GO TO SU.6**]
4 [VOL] Refuse [**GO TO SU.6**]

SU.2. How old were you when you first started smoking cigarettes (IF RESPONDENT REPORTS "DON"T KNOW" ASK HIM/HER TO PROVIDE THE BEST GUESS)?

_____AGE
[97 = 97+ YEARS OLD]
[98 = DON"T KNOW] [99 = REFUSE]

SU.3. Do you now smoke cigarettes every day, some days, or not at all?

1 Everyday
2 Some days
3 Not at all
4 [VOL] Don't know

5 [VOL] Refuse

SU.4. On average, when you smoked cigarettes, about how many cigarettes do you [did you] smoke per day? [PROBE: YOUR BEST ESTIMATE IS FINE] [A PACK=20]

____CIGARETTES PER DAY [0-60]

60 = 60 OR MORE
98 = DON"T KNOW
99 = REFUSE

SU.6. The next questions are about your use of alcoholic beverages, including beer, wine, wine coolers, and hard liquor like vodka, gin or whiskey. How old were you the very first time you ever drank an alcoholic beverage?

_____YEARS OLD
(IF VOL): "NEVER" .... 997 **GO TO LS.1**
DON'T KNOW.........................998
REFUSED................................999

SU .6a. When I use the word "drink" in the next questions, I mean either a glass of wine, a can or bottle of beer, or a shot or jigger of liquor either alone or in a mixed drink. How old were you when you first started drinking at least 12 drinks in a year?

IF "ALL MY LIFE" OR "AS LONG AS I CAN REMEMBER," PROBE: Was it before your teens? IF NO/DK, PROBE: Was it before your twenties?

_____YEARS OLD

BEFORE TEENS 12
BEFORE 20s............................19
AFTER 20s.............................. 20
(IF VOL): "NEVER" .... 997 **GO TO LS.1**
DON'T KNOW........................ 998
REFUSED 999

SU.7. Think about the past 12 months. In the past 12 months, how often did you usually have at least one drink - nearly every day, three to four days a week, one to two days a week, one to three days a month, or less than once a month?

NEARLY EVERY DAY .... ……1
3 - 4 DAYS PER WEEK .... ……2
1 - 2 DAYS PER WEEK .... …… ... 3
1 - 3 DAYS PER MONTH ……………………………….……4
LESS THAN ONCE A MONTH ……………………….…....5
(IF VOL) DID NOT DRINK IN PAST 12 MONTHS …….6 **GO TO SU.8a
DON'T KNOW ………………………………………….…...8
REFUSED ………………………………………….…….9

SU8. On the days you drank in the past 12 months, about how many drinks did you usually have per day?

___NUMBER OF DRINKS PER DAY

DON'T KNOW .... ……998
REFUSED ………………..999

SU.8a. Think about the time after your war zone service or peace-keeping service. In the 12 months after your war zone or peace-keeping service, how often did you usually have at least one drink - nearly every day, three to four days a week, one to two days a week, one to three days a month, or less than once a month?

NEARLY EVERY DAY 1
3 - 4 DAYS PER WEEK 2
1 - 2 DAYS PER WEEK 3
1 - 3 DAYS PER MONTH 4
LESS THAN ONCE A MONTH 5
(IF VOL) DID NOT DRINK IN 12 MONTHS AFTER WARZONE...6 **GO TO SU.13**
DON'T KNOW 8
REFUSED 9

SU8b. On the days you drank in the 12 months after your war zone service, about how many drinks did you usually have per day?

___NUMBER OF DRINKS PER DAY

DON'T KNOW 998
REFUSED 999

SU.12. And thinking back to the 12 months ***after*** your last war zone or peace-keeping service, how often did you have 6 or more alcoholic drinks on one occasion [IF MORE THAN ONE, REPORT THIS FOR THE MOST RECENT]?
(NOTE: A drink is 1 can or bottle of beer, 1 glass of wine, 1 can or bottle of wine cooler, 1 cocktail, or 1 shot of liquor.)

1 Never
2 Less than monthly
3 Monthly
4 Weekly
5 Daily or almost daily
6 Don"t know
7 Refused

SU.13. Thinking about just the past 30 days, on how many days did you drink any alcoholic beverages? [PROBE: BEST ESTIMATE IS FINE] (NOTE: A drink is 1 can or bottle of beer, 1 glass of wine, 1 can or bottle of wine cooler, 1 cocktail, or 1 shot of liquor.)

____DAYS USED OUT OF 30 [0-30]
[98 DON"T KNOW; 99 REFUSE]
[VOL] Did not drink (SKIP TO SU15)

[ASK SU.14, IF 1-30 DAYS IN SU.13]

SU.14. On the days when you drank alcohol over the past 30 days, on average, how many drinks did you have each day? [PROBE: YOUR BEST ESTIMATE IS FINE]
(NOTE: A drink is 1 can or bottle of beer, 1 glass of wine, 1 can or bottle of wine cooler, 1 cocktail, or 1 shot of liquor.)

____DRINKS per day [0-20]
[20= 20 or more; 98 DON"T KNOW; 99 REFUSE]
[CHECK POINT: IF SU7 = 6; SKIP TO SU20]

SU.15. Now, I have a few more questions about drinking alcoholic beverages over the past 12 months. At any time over the past 12 months, have you felt that you should cut down on your drinking?

1 Yes
2 No
3 Not sure
4 Refused
5 [VOL] Didn't drink in past 12 months, SKIP TO SU20

SU.16. And, at any time over the past 12 months, have people annoyed you by criticizing or complaining about your drinking?

1 Yes
2 No
3 Not sure
4 Refused
5 [VOL] Didn't drink in past 12 months, SKIP TO SU20

SU.19a. How often did you have a drink containing alcohol in the past year?

Never ---> (IF NEVER, SKIP TO SU.20) .
Monthly or less
2 to 4 times a month
3 or 4 times per week 4
or more times a week

SU.19b. How many drinks did you have on a typical day when you were drinking in the past year?

1 or 2
3 or 4
5 or 6
7 to 9
10 or more

SU.19c. How often did you have 6 or more drinks on one occasion in the past year?

Never
Less than monthly
Monthly
Weekly
Daily or almost daily

(LIFETIME STRESSORS)

LS1. Some people who have been through events that may be extraordinarily stressful, frightening or upsetting to almost anyone. Now, I am going to ask you about some of these events. Please tell me if any of the following events ever happened to you in your lifetime?

a. Natural disaster -
b. Serious accident
c. Combat or war zone experience
d. Being attacked with weapon
e. Being attacked without weapon
ee. Being held captive
f. Forced sexual contact

g. Situation in which was seriously injured/hurt
h. Situation in which feared might be seriously injured/killed
i. Seeing someone injured or killed or cause serious harm to someone
j. Other extremely stressful situation (MENTIONED)
Don’t know
Refused

OTHERS & RECORD EACH SEPARATELY FOR READ-IN LATER; **<u>MAKE SURE THAT THIS “OTHER” RESPONSE DOES *NOT* DUPLICATE LS1a-LS1j ABOVE, PROBE IF NEEDED</u>**]

____[OPEN-END]

LS3. INTERVIEWER CHECK POINT: SEE LS1a – LS1j <u>AND</u> LS2 [OPEN-END QUESTION]

LS1a – LS1j: TWO OR MORE CODED „1‟ …………...……...……….1 **GO TO LS4**
LS1a – LS1j: ONE CODED „1‟ & ONE EVENT MENTION IN LS2……2 **GO TO LS4**
LS1a – LS1j: NONE CODED „1‟ AND >1 EVENT MENTION IN LS2…3 **GO TO LS4**
ALL OTHER:; ONE TOTAL STRESSOR ……………………......…..4 **GO TO LS5**
ALL OTHERS: ZERO TOTAL STRESSORS……………………......5 **GO TO CH1**

LS4. Of the stressful events just mentioned, which <u>one</u> affected you the <u>most</u>?
a. Natural disaster…………………………………………………1
b. Serious accident ………………………………………………..2
c. Combat or war zone experience…………………………………. .3
d. Being attacked with weapon ……………………………………...4
e. Being attacked without weapon……………………………………5
ee. Being held captive..6
f. Forced sexual contact…………………………………………....7
g. Situation in which was seriously injured/hurt……………………..8
h. Situation in which feared might be seriously injured/killed………9
i. Seeing someone injured or killed…………………………………..10
ii. Caused serious harm/injury to someone..11
j. Other extremely stressful situation (MENTIONED)……………..12
Don’t know……………………………………………………..98
Refused…………………………………………………………99

LS5. During this <u>most stressful</u> event [IF MORE THAN ONE EVENT REPORTED], did you…

	Yes	No	DK	Refused
a. actually experienced this event?..…………………….....	1	2	8	9
b**. or** only witnessed this event?….......…………………	1	2	8	9

LS6. INTERVIEWER CHECK POINT: SEE LS1a – LS1j <u>AND</u> LS2 [OPEN-END QUESTION]
.
LS1a – LS1j: TWO OR MORE CODED „1‟ …………...……...……….1 **GO TO LS7**
LS1a – LS1j: ONE CODED ‘1’ & ONE EVENT MENTION IN LS2……2 **GO TO LS7**

ALL OTHERS………………………………………………………...4 **GO TO CH1**

LS7. Out of the multiple stressful events mentioned, you had mentioned one event that affected you the most. Out of the multiple events experienced, what event affected you the <u>second most</u>?

a. Natural disaster……………………………………………………1
b. Serious accident ……………………………………………………...2
c. Combat or war zone experience………………………………….. 3
d. Being attacked with weapon ……………………………………...4
e. Being attacked without weapon…………………………………5
ee. Being held captive 6
f. Forced sexual contact…………………………………………. .7
g. Situation in which was seriously injured/hurt……………………...8
h. Situation in which feared might be seriously injured/killed……….9
i. Seeing someone injured or killed………………………………..10
ii. Caused serious harm/injury to someone 11
j. Other extremely stressful situation (MENTIONED)……………...12
k. None ………………………………………………………97 **GO TO CH1**
Don"t know……………………………………………………..98 **GO TO CH1**
Refused…………………………………………………………99 **GO TO CH1**

LS8. During this second most stressful event [IF MORE THAN ONE EVENT REPORTED], did you…

	Yes	No	DK	Refused
a. actually experienced this event?..…………………....1	1	2	8	9
b. **or** only witnessed this event?…......……………..……..1	1	2	8	9

(CHILDHOOD EVENTS)

CH1. The following relates to events that could have happen to you before you were 18 years old. Please tell me if the following events never, sometimes, often, or very often happened before you were 18 years old?	**NEVER (1)**	**SOME-TIMES (2)**	**OFTEN (3)**	**VERY OFTEN (4)**	**DK (8)**	**REF (9)**
CH1a. Did a parent or other adult in the household ever swear at you, insult you or put you down?	1	2	3	4	8	9
CH1b. Did a parent or other adult in the household ever act in a way that made you afraid that you might be physically hurt?	1	2	3	4	8	9
CH1c. Would you say that your family was a source of strength or support?	1	2	3	4	8	9
CH1d. Did a parent or other adult in the household ever push, grab, slap, or throw something at you?	1	2	3	4	8	9

CH1e. Did a parent or other adult in the household ever hit you so hard that you had marks or were injured?	1	2	3	4	8	9
CH1f. In your household, was your mother or stepmother ever pushed, grabbed, slapped, or had something	1	2	3	4	8	9
CH1g. Was there someone in your family that helped you feel special or important?	1	2	3	4	8	9
CH1h. In your household, was your mother or stepmother ever hit, kicked, punched, or hit with something hard?	1	2	3	4	8	9
CH1i. Did your ever have to wear dirty clothes?	1	2	3	4	8	9
CH1j. Did you go hungry or not have enough to eat in your household?	1	2	3	4	8	9
CH1k. Would you say that there was someone to take care of you and protect you?	1	2	3	4	8	9
CH1l. Were your parents or step-parents too high or drunk to take care of you?	1	2	3	4	8	9

(DEPRESSION)

MD1. Now, let's talk about some specific complaints that people sometimes have…
Have you ever had a period of two weeks or longer when you were feeling depressed or down most of the day or nearly everyday?

1 Yes
2 No
8 Don‟t know
9 Refuse

MD2. What about a time of two weeks or longer when you were uninterested in most things or unable to enjoy things you used to do?

1 Yes
2 No
3 Don‟t know
4 Refuse

MD3. Have you ever had a period of two weeks or longer when you [READ EACH ITEM]

**[ROTATE ITEMS]**	Yes	No	Don‟t know	Refuse
a. Lost or gained weight without dieting?	1	2	8	9
b. Slept too much, too little or a lot less than normal for you?	1	2	8	9
c. Were so fidgety or restless that you were unable to sit still?	1	2	8	9
**Continue Scale Below:**				

d. Were talking or moving more slowly than is normal for you?	1	2	8	9
e. Felt tired all of the time or low in energy all of the time?	1	2	8	9
f. Felt worthless or felt guilty about things that you had or had not done in the past?	1	2	8	9
g. Had a hard time thinking, or concentrating or making decisions about everyday things?	1	2	8	9
h. Felt that things were so bad that you thought about hurting yourself or that you'd be better off dead?	1	2	8	9

[INTERVIEWER: IF PATIENT ANSWERS „YES‟ TO MD3h, THEN OFFER INFORMATION ON COUNSELING SERVICES]

MD4. INTERVIEWER CHECKPOINT: (SEE MD1, MD2, MD3a – MD3h)

< 3 RESPONSES CODED '1' …..…..……..1 **GO TO MD11**
ALL OTHERS ……………………………..2

MD5. Did these experiences, moods, or feelings you've had ever seem to go together or happen at the same time--at least within the same month?

1 Yes
2 No **[GO MD11]**
8 Don't know
9 Refuse

MD6. When you had these experiences or feelings together or happen at the same time, at least within the same month, did these only happen when you had a medical problem, because of a death in the family, or because of a problem with alcohol or drugs?

1 Yes [**GO MD11**]
2 No, happened at other times
8 Don't know
9 Refuse

MD7. When was the most recent time you had a lot of these experiences, moods or feelings at the same time? [READ CHOICES]

1 Was it within the past month,
2 Was it within the past 6 months,
3 Within the past year,
4 or, more than 1 year ago?
8 Don't know [VOL]
9 Refuse [VOL]

MD8. And, when you recently had a lot of these experiences, moods or feelings at the same time, how difficult did these problems made it for you to work, take care of things, get along with people, or to function? [READ CHOICES]

1 Not difficult at all,
2 Somewhat difficult,
3 Very difficult,
4 or, Extremely difficult?
8 Don't know [VOL]
9 Refuse [VOL]

MD9. How old were you, the first time you first had these experiences, moods or feelings at the same time (IF RESPONDENT REPORTS “DON‟T KNOW” ASK HIM/HER TO PROVIDE THE BEST GUESS) ?

______[AGE]
[998 = DON‟T KNOW; 999 = REFUSE]

MD10. And, the first time had a lot of these experiences, moods or feelings at the same time, how difficult did these problems made it for you to work, take care of things, or get along with people? [READ CHOICES]

1 Not difficult at all,
2 Somewhat difficult,
3 Very difficult,
4 or, Extremely difficult?
8 Don‟t know [VOL]
9 Refuse [VOL]

(SUCIDE)

MD11. Have any of the following experiences **ever** happen to you?

a. You seriously thought about committing suicide?
b. You made a plan for committing suicide?
c. You attempted suicide?

[INTERVIEWER: IF PATIENT ANSWERS „YES‟ TO MD9a-c, THEN OFFER INFORMATION ON COUNSELING SERVICES]

(PTSD)

PT1. People sometimes have a response to a very stressful experience. As I read each item, please indicate how much you have been bothered by that problem in the **past 12 months**... [READ ITEM]

PT1a. **[FOR EACH ITEM MENTIONED IN PT1, ASK PT1a]** How much you have been bothered by that problem within the **past 30 days**?

PT2. **[FOR EACH PROBLEM MENTIONED IN PT1/PT1a, ASK PT2, WHERE APPROPRIATE]** Was that related to the most stressful event mentioned earlier [IN LS4], the second most stressful event mentioned [IN LS7], or another event [CODE ALL THAT APPLY]?

	PT1	PT1a	PT2 - EVENT				
[DO NOT ROTATE] [ONLY ASK PT1a QUESTIONS, IF PT BOTHERED A LITTLE BIT OR MORE IN PT1] -------------------------->	**Not at all=0<br>A little bit=1<br>Moderately=2<br>Quite a bit=3<br>Extremely=4**	**Not at all=0<br>A little bit=1<br>Moderately=2<br>Quite a bit=3<br>Extremely=4**	**Most Stress**	**2nd Most Stress**	**Other Event**	**More than 1**	**[VOL] None/DK**
1. Repeated, disturbing, and unwanted memories of the stressful experience?	0 1 2 3 4	0 1 2 3 4	1	2	3	4	5
2. Repeated, disturbing dreams of the stressful experience?	0 1 2 3 4	0 1 2 3 4	1	2	3	4	5
3. Suddenly feeling or acting as if the stressful experience were actually happening again (as if you were actually back there reliving it)?	0 1 2 3 4	0 1 2 3 4	1	2	3	4	5
4. Feeling very upset when something reminded you of the stressful experience?	0 1 2 3 4	0 1 2 3 4	1	2	3	4	5
5. Having strong physical reactions when something reminded you of the stressful experience (for example, heart pounding, trouble breathing, sweating)?	0 1 2 3 4	0 1 2 3 4	1	2	3	4	5
6. Avoiding memories, thoughts, or feelings related to the stressful experience?	0 1 2 3 4	0 1 2 3 4	1	2	3	4	5
7. Avoiding external reminders of the stressful experience (for example, people, places, conversations, activities, objects, or situations)?	0 1 2 3 4	0 1 2 3 4	1	2	3	4	5
8. Trouble remembering important parts of the stressful experience?	0 1 2 3 4	0 1 2 3 4	1	2	3	4	5
9. Having strong negative beliefs about yourself, other people, or the world (for example, having thoughts such as: I am bad, no one can be trusted, the world is completely dangerous)?	0 1 2 3 4	0 1 2 3 4	1	2	3	4	5
10. Blaming yourself or someone else for the stressful experience or what happened after it?	0 1 2 3 4	0 1 2 3 4	1	2	3	4	5
11. Having strong negative feelings such as fear, horror, anger, guilt, or shame?	0 1 2 3 4	0 1 2 3 4	1	2	3	4	5
12. Loss of interest in activities that you used to enjoy?	0 1 2 3 4	0 1 2 3 4	1	2	3	4	5
13. Feeling distant or cut off from other people?	0 1 2 3 4	0 1 2 3 4	1	2	3	4	5
14. Trouble experiencing positive feelings (for example, being unable to feel happiness or have loving feelings for people close to you)?	0 1 2 3 4	0 1 2 3 4	1	2	3	4	5
15. Irritable behavior, angry outbursts, or acting aggressively?	0 1 2 3 4	0 1 2 3 4	1	2	3	4	5
16. Taking too many risks or doing things that could cause you harm?	0 1 2 3 4	0 1 2 3 4	1	2	3	4	5
17. Being "super-alert" or watchful or on guard?	0 1 2 3 4	0 1 2 3 4	1	2	3	4	5
18. Feeling jumpy or easily startled?	0 1 2 3 4	0 1 2 3 4	1	2	3	4	5
19. Having difficulty concentrating?	0 1 2 3 4	0 1 2 3 4	1	2	3	4	5
20. Trouble falling or staying asleep?	0 1 2 3 4	0 1 2 3 4	1	2	3	4	5

Continue PTSD Question with PT4 Below.

PT4. INTERVIEWER CHECKPOINT: (SEE: PT1 to PT20 for Past Year)
2+ POSITIVE RESPONSES MENTIONED …..…..………..…..1
ALL OTHERS ………………………….....................……….2 GO TO PT12

PT5. You said that you have had some problems concerning moods and feelings following a most stressful event
[MOST STRESSFUL EVENT MENTIONED IN LS1, LS2 OR LS4] or another highly stressful event mentioned [IF LS3 = 1, 2, OR 3]. Did several of these problems, moods or feelings you mentioned ever seem happen at the same time, at least within the same month following these events?

1 Yes
2 No [GO TO PT12]
8 Don't know [GO TO PT12]
9 Refuse [GO TO PT12]

PT6 What is the longest period of time that you had several of these problems, moods, feelings at the same time following a most stressful event [MOST STRESSFUL EVENT MENTIONED IN LS1, LS2 OR LS4], or another stressful event mentioned [IF LS3 = 1, 2, OR 3]? Was it for… [READ LIST]

1 Less than one month,
2 One to three months, OR
3 More than three months?
8 [VOL] Don‟t know
9 [VOL] Refused

PT8. When was the last time you had several of these problems, feelings or moods at the same time related to a most stressful event [MOST STRESSFUL EVENT MENTIONED IN LS1, LS2 OR LS4] or another most stressful event mentioned [IF LS3 = 1, 2, OR 3]? Was it: [READ CHOICES]

1 Within the past 30 days,
2 Within the past 6 months,
3 About 1 year ago,
4 Or more than a year ago?
8 Don‟t know [VOL]
9 Refuse [VOL]

PT9 How old were you, the first time you had these experiences, moods or feelings at the same time related to a most stressful event [MOST STRESSFUL EVENT MENTIONED IN LS1, LS2 OR LS4], or another stressful event mentioned [IF LS3 = 1, 2, OR 3]?

[AGE]

[998 = DON"T KNOW; 999 = REFUSE]

PT10. When you had several of these problems, feelings or moods at the same time related to a most stressful event [MOST STRESSFUL EVENT MENTIONED IN LS1, LS2 OR LS4] or another most stressful event mentioned [IF LS3 = 1, 2, OR 3], how difficult did these problems make it for you to work, take care of things, get along with people, or to function? [READ CHOICES]

1 Not difficult at all,
2 Somewhat difficult,
3 Very difficult,
4 or, Extremely difficult?
8 Don't know [VOL]
9 Refuse [VOL]

[IF P2_1-P2_20 = 2+ FOR PT1 (PAST YEAR) I.e., Moderate, Quite a bit or Extreme for 2 or more items go to PT11, OTHERWISE SKIP TO PT12]

PT11. You mentioned you were exposed to "other" stressful events. During this "other" event(s), did you…

	Yes	No	DK	Refused
a. Actually experienced this event(s)?…………….	1	2	8	9
b. Or only witnessed this vent(s)?........………….	1	2	8	9

PT12. Over the past 12 months, how difficult was it for you to fall asleep?

Very difficult...……………………………………………1
Difficult……...…………………………………………….2
Somewhat difficult ….…………………………………….3
Not difficult...………………………………………… 4
Not difficult at all...….……………………………………5
Don't know [VOL] ...……………………………………..8
Refuse [VOL]……………………………………………..9

PT13. Over the past 12 months, how difficult was it for you to stay asleep?

Very difficult...……………………………………………1
Difficult……...…………………………………………….2
Somewhat difficult ….…………………………………….3
Not difficult...……………………………………......….4
Not difficult at all...……………………………………….5
Don't know [VOL] ...……………………………………..8
Refuse [VOL]……………………………………………..9

SS1. Did you ever have a period lasting six months or longer during your childhood or adolescence when you frequently did things that got you in trouble with adults such as losing your temper, arguing or talking back to adults, refusing to do what your teachers or parents asked you to do?

YES ....………………………............. 1
NO ....……………………… 2
DON"T KNOW ....……………………… 8
REFUSED ....……………………… 9

SS2. Many children and teenagers go through periods when they do things adults don't want them to do, like lying, stealing, or breaking rules. Did you ever go through a period during your childhood or teenage years when you did any of these things?

YES ..………………………................................ 1
NO ....……………………… 2
DON"T KNOW ....……………………… 8
REFUSED ....……………………… 9

SS3. Did you ever go through a period as a child or teenager when you either broke into cars, set fires, or destroyed property on purpose?

YES ....…………………….............. 1
NO ....……………………......2
DON"T KNOW ....…………..……… 8
REFUSED ....……………………........9

SS4. When you were a child or a teenager, did you ever run away from home, or repeatedly play hooky from school, or often stay out much later at night than you were supposed to?

YES ....…………………… 1
NO ....……………………......2
DON"T KNOW ....…………………….. 8
REFUSED.....................…………………….. 9

(PERSONALITY)

SS11. Here are a number of traits that may or may not apply to you. Please write a number next to each statement (A through J) to indicate the extent to which you agree or disagree with that statement for you.

**Disagree strongly**	**Disagree moderately**	**Disagree a little**	**Neither agree nor disagree**	**Agree a little**	**Agree moderately**	**Agree strongly**
1	2	3	4	5	6	7

I see myself as:

1. ____ Extraverted, enthusiastic.
2. ____ Critical, quarrelsome.
3. ____ Dependable, self-disciplined.
4. ____ Anxious, easily upset.
5. ____ Open to new experiences, complex.
6. ____ Reserved, quiet.
7. ____ Sympathetic, warm.
8. ____ Disorganized, careless.
9. ____ Calm, emotionally stable.
10. ____ Conventional, uncreative.

(DEMOGRAPHICS)

Now I am going to ask you some demographic and other information….

D.1. The next few questions are for background purposes. Are you currently married, separated, divorced, widowed or never married?

MARRIED .... 1 **GO TO D.3**
SEPARATED 2
DIVORCED.. 3
WIDOWED 4
NEVER MARRIED.. 5
DON"T KNOW.. 8
REFUSED.. 9

D.2. Are you currently living with someone in a marriage-like relationship?

YES... 1
NO... 2
DON'T KNOW 8
REFUSED 9

D.2a. How many children do have? _____

D.3. How tall are you?.....................____(FEET)_______________(INCHES)

DON"T KNOW.....................998
REFUSED..............................999

D.4. **[LBS]** How much do you weigh?_________(POUNDS)

DON"T KNOW....................998
REFUSED............................ 999

D.5. What is the highest level of education or schooling you completed? READ CATEGORIES AS NEEDED]

1	Never attended school
2	Kindergarten to 8th grade
3	Some high school (9th to 11th grade)
4	High school equivalency (GED)
5	High school graduate (12th grade)
6	Some college, community college or technical training
7	College graduate (4 Year Degree Program)
8	Graduate work
9	Don't know
10	Refuse

D.6. What was your total combined household income last year? Include your income and income for anyone else living in your household.

[PROBE: Your best estimate is fine.] READ CATEGORIES TO PROBE: Would it be:]

Under $20,000 1
$20,000-to just under $30,000 2
$30,000-to just under $40,000 3
$40,000-to just under $50,000 4
$50,000-to just under $75,000 5
$75,000 to just under $100,000....................6
$100,000 or over..7
VOL Not sure 8
VOL Refused 9

D7. How old are you? ____

D8. Record respondent's gender (DO NOT ASK):

Male 1
Female 2

D11 Are you of Spanish or Hispanic origin?

Yes
No
Don't know
Refuse

D13. How would you describe your racial background? Would you describe yourself as: [READ CHOICES, MULIPLE CHOICES ALLOWED]

White,
Black or African American,
Asian,
Native Hawaiian or Other Pacific Islander, or
American Indian or Alaska Native?
[VOL] Other [SPEC________________]
[VOL] Hispanic
[VOL] None of these
[VOL] Don't know
[VOL] Refuse

DNA2. The packet will include: two consent forms (one should be kept as your copy), a saliva collection kit with instructions, and a self-addressed stamped return envelope. The materials that we ask for in return are one signed consent form and the saliva kit. A second gift card for $15 will be mailed to you once we receive these completed materials.

QUESTIONS TO ASSESS NEED FOR COUNSELING SERVICES

That brings us to the end of the survey, but I have a couple of things to take care of before I go.

C1. Some of the questions in this survey can be upsetting or stressful. Were any of the survey questions emotionally upsetting to you?

1 Yes [ASK **C2**]
2 No [GO TO **C6**]

C2. Before I go, I just want to be sure that you are feeling okay. Are you still feeling emotionally upset or are you okay now?

1 Still upset [ASK **C3**]
2 Feeling okay now [GO TO **C6**]

C3. If you would like to speak to someone about how you are feeling, I can provide you with information about counseling services.

1 Yes, would like info about counseling services [ASK **C4**]
2 No, do not want counseling services [GO TO **C6**]

C4. If you need someone to call you right away I can arrange for a doctor/counselor to get back to you. If you do not need someone to call you right away, I can give you a toll free number to call when you're ready to speak to someone about counseling services.

1 Need someone to call right away [GO TO **C5** AND FILL OUT INFO REQ.]
2 Do NOT need immediate assistance - get 800 number [GO TO **C6**]

C5. RESPONDENT CONTACT INFORMATION

What is your name/address? ________________________________

And, where do you live?____(I need to provide the Doctor/counselor with your name, address and telephone - we do not keep the information. Your name and address are never associated with your survey answers.)

Interviewer Comments: ____

"Okay, I'll have someone get back to you soon."

[IF DOES NOT NEED IMMEDIATE ASSISTANCE - GET TOLL FREE NUMBER & STUDY REFERRAL LIST; MAKE SURE YOU HAVE CURRENT PSYCHIATRY REFERRAL LISTS]

C6. As I mentioned before, we have contact information that people can call to arrange for local counseling services. We can send you this information if you'd like.

1 Accepted information
2 Does not want information

NOTE: For PTSD positive status, count Q1-Q20 for past year; if 6 or more are positive (i.e., moderate, quite a bit, or extremely), offer a referral for respondent.

NOTE: For Depression positive status, IF MD4 CHECKPOINT = 2 (I.E., 3+ SYMPTOMS CODED "YES" [CODE = 1] FOR MD1, MD2, MD3a - MD3h) AND MD7 is coded 1, 2, OR 3, offer a referral for respondent.

CONCLUSION

[ASK ALL]

C8. Now, I would just like to give you our telephone number in case you have any additional questions about the project or you need to get in touch with us later on. Would you like to write our number down? (toll free telephone number is 1-866-TBD)

1 Accepts number
2 Did not want the number

Thank you for your participation and your US military service!
