# Supplementary material for: Risk and protective factors associated with mental health among female military veterans: results from the veterans’ health study
Source: BMC Womens Health. 2021 Feb 8;21:55. doi: 10.1186/s12905-021-01181-z (PMC7869200; doi:10.1186/s12905-021-01181-z)
Supplement: Supplementary file 2 — Additional file 2. Survey script. [file 12905_2021_1181_MOESM2_ESM.pdf]

Veteran Status Questionnaire

Name[redacted]

In order to better serve healthcare needs, the Geisinger Health System is incorporating veteran status into its healthcare records. We have a few questions to ask you. **Please return to receptionist when completed.**

Please complete one of the sections below for EACH period you served on active duty or were deployed in the US Armed Services.

Most Recent Period

1. Served from: ______________ (Month/Year) Served to: ______________(Month/Year)

2. What service branch did you serve in during this period of active duty or deployment? (check one)

   [ ] US Army [ ] US Navy [ ] US Marines [ ] US Air Force [ ] US Coast Guard [ ] Unknown

3. During this period of active duty or deployment were you a member of the National Guard or the Reserves?

   [ ] Yes [ ] No [ ] Unknown

4. Did you serve in a war zone or see combat during this period of active duty or deployment?

   [ ] Yes [ ] No [ ] Unknown

5. If "Yes" to question 4, which combat or war zone did you serve in during this active duty or deployment?

   [ ] World War II in Pacific or Europe (1940-1946) [ ] Korea (1950-1955) [ ] Vietnam (1960-1975)
   [ ] Persian Gulf (1990-1992) [ ] Iraq/Afghanistan (2001-2009) [ ] Other: ______________
   [ ] Unknown

Second Most Recent Period (If applicable)

1. Served from: ______________ (Month/Year) Served to: ______________(Month/Year)

2. What service branch did you serve in during this period of active duty or deployment? (check one)

   [ ] US Army [ ] US Navy [ ] US Marines [ ] US Air Force [ ] US Coast Guard [ ] Unknown

3. During this period of active duty or deployment were you a member of the National Guard or the Reserves?

   [ ] Yes [ ] No [ ] Unknown

4. Did you serve in a war zone or see combat during this period of active duty or deployment?

   [ ] Yes [ ] No [ ] Unknown

5. If "Yes" to question 4, which combat or war zone did you serve in during this active duty or deployment?

   [ ] World War II in Pacific or Europe (1940-1946) [ ] Korea (1950-1955) [ ] Vietnam (1960-1975)
   [ ] Persian Gulf (1990-1992) [ ] Iraq/Afghanistan (2001-2009) [ ] Other: ______________
   [ ] Unknown

Third Most Recent Period (If applicable)

1. Served from: ______________ (Month/Year) Served to: ______________(Month/Year)

2. What service branch did you serve in during this period of active duty or deployment? (check one)

   [ ] US Army [ ] US Navy [ ] US Marines [ ] US Air Force [ ] US Coast Guard [ ] Unknown

3. During this period of active duty or deployment were you a member of the National Guard or the Reserves?

   [ ] Yes [ ] No [ ] Unknown

4. Did you serve in a war zone or see combat during this period of active duty or deployment?

   [ ] Yes [ ] No [ ] Unknown

5. If "Yes" to question 4, which combat or war zone did you serve in during this active duty or deployment?

   [ ] World War II in Pacific or Europe (1940-1946) [ ] Korea (1950-1955) [ ] Vietnam (1960-1975)
   [ ] Persian Gulf (1990-1992) [ ] Iraq/Afghanistan (2001-2009) [ ] Other: ______________
   [ ] Unknown

Thank you very much for providing this information and for your service to the country. This information will be helpful in better serving your healthcare needs.
